# Lead, Zinc, Copper, and Cadmium Content of Water from South Australian Rainwater Tanks

**DOI:** 10.3390/ijerph15071551

**Published:** 2018-07-23

**Authors:** Chirhakarhula E. Chubaka, Harriet Whiley, John W. Edwards, Kirstin E. Ross

**Affiliations:** Environmental Health, College of Science and Engineering, Flinders University, GPO Box 2100, Adelaide 5001, Australia; Harriet.Whiley@flinders.edu.au (H.W.); John.Edwards@flinders.edu.au (J.W.E.); Kirstin.Ross@flinders.edu.au (K.E.R.)

**Keywords:** public health, potable water, rainwater, metals, lead, zinc, copper, cadmium

## Abstract

Rainwater is consumed for drinking water in many parts of Australia, either preferentially over municipal water or in regional or remote areas, because rainwater is the primary source of water. Previous rainwater studies in other areas in Australia have shown the levels of some metals to be above the Australian Drinking Water Guidelines (ADWG). This study assessed the level of metals in rainwater harvested in the Adelaide region. Water samples were collected from 53 tanks from three different sampling corridors. A total of 365 water samples were analysed for lead, zinc, copper, and cadmium using atomic absorption spectrophotometry. In 47 out of the 53 tanks, lead was above the ADWG of 0.01 ppm in at least one sample (with 180/365 samples above 0.01 ppm). Zinc was above the ADWG (3.0 ppm) in 53/365 samples, copper was above the ADWG (2.0 ppm) in eight samples out of 365 samples, and cadmium was above the ADWG (0.002 ppm) in 19 samples out of 365 samples. These data are consistent with other studies of rainwater quality in Australia. Comparisons of levels of metals and volume of rainfall in the sampling and preceding month, roof material, and tank material, the presence of a first-flush device, sampling corridor, and sample pH showed that the roof material was related to higher levels of metals. There was a significant relationship between sampling corridors and the levels of lead and zinc. Nine of the tanks surveyed had filters installed. There was a small, but statistically significant, decrease in the levels of metals that passed through a filter prior to collection but, in those samples, filters did not remove metals to below guideline concentrations. An estimate of exposure, and a brief discussion of health risks as a result of exposure to metals, is presented.

## 1. Introduction

In Australia, rainwater is used as a potable and non-potable water source [1]. In some cases it is used for drinking water preferentially to the municipal water supply, and in other cases it is the only available domestic water source [2]. There is a general consensus in the community that drinking rainwater is safe for human consumption [3]. However, research indicates that roof-harvested rainwater can contain bacteria and metals above the Australian Drinking Water Guideline (ADWG) limits [4]. The presence of elevated levels of metals, such as lead, cadmium, and copper, can present an issue of concern for human health [5,6,7]. Lead is a cumulative toxin that affects the central nervous system and can trigger the dysfunction of renal and cardiovascular systems [8,9,10]. Lead can also affect brain development and impact on the human intellectual quotient (IQ) [11]. Cadmium components are classified as a Group 1 carcinogen [12] and long-term exposure has the potential to affect reproductive organs, cause low birth weights, kidney damage, and cardiovascular or nervous system impairment [13,14,15].

In low concentrations, copper is an essential trace element that play a role in many enzymes involved in vital biological processes. However, chronic exposure or high concentrations causes oxidative stress resulting in kidney, gastrointestinal tract, or liver damage, which can be fatal [16,17]. At high intake, copper is classified Group 3 human carcinogen [12]. In drinking water, copper gives the water an unpleasant taste at 3 mg/L and, in susceptible people, illness incidents can occur from a concentration of 2 mg/L [18]. Comparatively, zinc is less hazardous to human health and zinc deficiency has the potential to impact human growth, neuronal development and the immune system [19]. In drinking water, however, zinc can cause a metallic taste to water at concentrations above 3.0 mg/L [18]. 

Unlike municipal water, which is subject to treatment to ensure it is safe for human consumption, rainwater is harvested and consumed untreated by many households [20]. Previous studies have found that rainwater collected in Australia contained trace metals, but generally within health guidelines [21]. However, in urban Australia and in former industrial and mining precincts, metals such as lead, chromium, silver, nickel, copper, arsenic, and manganese have been detected in rainwater above health limits [22,23,24]. This study investigated the concentrations of lead, zinc, copper, and cadmium in samples sampled from three sampling corridors around Adelaide; an urban area, a peri-urban area, and a rural area outside of Adelaide. The effect of the volume of rainfall in the sampling and preceding month, roof material, tank material, and the presence of a first-flush device, sampling corridor, and sample pH was examined. The effect of filtration was also examined in those homes that had a filter fitted to their rainwater tank.

## 2. Material and Methods

### 2.1. Study Site

**Figure 1:**
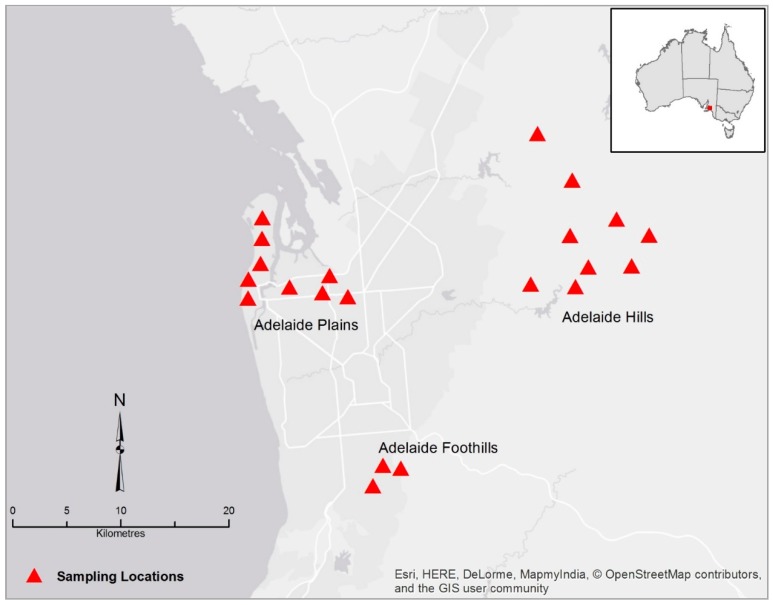
Study area, Adelaide region. The geographical spread of a study corridor is illustrated by the number Δ, this does not correlate to number of samples. ESRI Map Data, Delorme maps 2018.

Rainwater samples were collected in the Adelaide region nine times over a fourteen-month period that covered more than one year from July 2015 to August 2016. The study was approved by Flinders University Social and Behavioural Research Ethics Committee (SBREC No. 6782) in accordance with the National Statement on Ethical Conduct in Human Research (NSECHR). The selection of sampling corridors was based on land use, and on vegetation cover. Three sampling corridors were selected in the Adelaide region, based on vegetation cover and on human activities. One sampling corridor, predominantly residential with light manufacturing industries, was selected in the Adelaide plains (urban corridor). The second corridor, essentially residential with high vegetation cover was selected in the Adelaide foothills (peri-urban corridor). The third corridor was selected in semi-rural suburbs in the Adelaide hills that spreads from One Tree Hill to Paracombe, Kersbrook, Gumeracha, and Cuddle Creek (rural corridor) (Figure 1).

Households approached to participate in the study were randomly chosen based on the criteria that they were located within a sampling corridor and had a rainwater tank. The initial contact with tank owners was through direct contact (knocking on doors). The decision to participate to the study was voluntary. A briefing on the study objectives was given to tank owners before they made their decision to participate. A letter of introduction, a consent form and a questionnaire for the study were given to every household. Questions were about their water tank, whether they had first flush devices, and questions about what the water was used for, whether their tanks had filters fitted and whether the tanks were plumbed directly into the houses. Additional details on tank and roof structure materials were taken by looking at the tank and roof structure. Metals roofs were grouped together (colorbond^®^ steel, zincalume^®^, galvanised iron, etc.).

### 2.2. Sampling Techniques

The water was collected in 1 L acid washed polyurethane bottles. During transport from the field to the laboratory, all samples containers were tightly closed and properly labelled, to avoid incidents of sample cross-contamination. There were zero detections in many samples indicating that there was no leaching from the equipment used to collect and transport the samples. During sample collection, the water was run for several seconds before collecting. Then, the samples were transported back to the laboratory on ice and processed immediately on arrival. The samples were stored refrigerated and acidified with 2.5 mL nitric acid in a 2.5 mL polyurethane container. The acidified samples were stored at 4 °C prior to testing. Water parameters such as water pH and water temperature were taken in the field. A total of 365 rainwater samples were collected across these three corridors from 53 tanks, with 120 samples collected in the Adelaide plains from 18 tanks, 97 samples in the Adelaide foothills from 15 tanks, and 148 samples in the Adelaide Hills from 20 tanks.

Samples were collected every month, or after a significant storm that occurred between two scheduled sampling dates. Many tanks did not have water for sampling in summer, after drier conditions that prevail in the Adelaide region in summer months and so were not sampled. Nine (16.9%) of the tanks surveyed had filters installed. These tanks were sampled before the filter using the method described above, and a second sample taken post-filtration at the tap inside the house. These pre-filtration samples were included in all the analyses (365 samples), but the post-filtration sample was not. 

### 2.3. Samples Preparation, Testing, and Analysis

All samples were filtered prior to analysis through 22 µm pore size filters (Whatman^®^ ashless filters Grade 541, Whatman, London, UK) to reduce the volume of suspended solids associated with rainwater, to minimise risks of obstruction on the instrument capillary tubing. Paper filters can contain metals that can be leached out in aqueous matrices during water filtration [25,26]. Therefore, blanks using filters were created. Sample analysis was carried on a Grey-Bartlett-Charlton 933 atomic absorption spectrometer unit (GBC 933 AAS) (GBC Scientific Equipment Australia, Dandenong, Melbourne, Australia). Cableless Photron Coded Hollow Cathode Lamps for common elements were used to source radiation to excite the free atoms into the atomic flame (Photron Ptd Ltd., Narre Warren, Victoria, Australia) [27]. The GBC 933 AAS uses air and acetylene as fuel to ignite the atomic flame [28]. Avanta Software for Windows^®^ 95 (GBC Scientific Equipment Pty Ltd., Dandenong, Victoria, Australia) was used for the GBC 933 AAS calibration and data processing. Lead, zinc, cadmium, and copper stock solutions were created using 2% nitric acid (HNO_3_). A volume of 1.12 mL nitric acid (HNO_3_) at 1% concentration was added to 100 mL deionised water to create calibration blanks which were run in between every sample. All samples were analysed in triplicate. Metals were determined as total metals. The GBC 933 AAS elemental detection limits were 0.009 mg/L for cadmium, 0.025 mg/L for copper, 0.06 mg/L for lead, and 0.008 mg/L for zinc.

### 2.4. Data Analysis

All data were entered and graphed using Microsoft Excel (Microsoft Corporation, Washington, DC, USA) and GraphPad Prism software (GraphPad Software, Inc., San Diego, CA, USA). Data were tested for normality and an analysis of variance of metal concentrations and different roof and tank material, the presence of a first flush device, sampling corridor, and pH was conducted. Before and after filtration metal concentration was compared using a paired *t*-test.

## 3. Results

### 3.1. Metal Levels Detected above the ADWG

Metals of health concerns and/or unacceptable for water drinking water taste were detected in tanks at least once over the ADWG values. Lead was the metal detected most often above the ADWG (180/365 samples). In total, 47 tanks (88.6%) were positive for lead above the ADWG guideline of 0.01 ppm at least once and the highest concentration detected was 3.24 ppm. Higher lead levels were detected most often in the Adelaide foothills. In that area, 51% of samples contained lead above the ADWG, compared with 41% in the Adelaide hills, and 27% in the Adelaide plains (Table 1). In total, 29 samples (7.9%) that exceeded the guidelines of 0.01 mg/L were taken from tanks that used the water for drinking.

**Figure 2:**
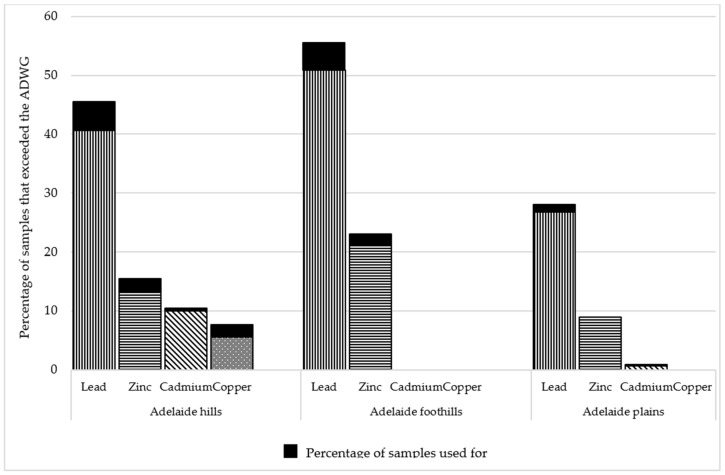
The percentage of total samples and samples used for drinking above the ADWG for each sampling location.

In addition to lead, the study detected zinc, cadmium, and copper in a few samples that exceeded the ADWG limits. Zinc was above the ADWG of 3.0 ppm in 53 (14.5%) samples. As with lead, the number of samples that exceeded the zinc ADWG limits was highest in the Adelaide foothills, and lowest in the Adelaide plains (Figure 2). It should be noted that the zinc and copper guidelines are guidelines for aesthetic reasons and not health based guidelines [18]. Cadmium and copper were also detected in a few samples. Cadmium was detected in 19 samples (5.2%) above the guideline of 0.002 ppm. The detection of copper above the ADWG of 2.0 ppm was limited to eight samples (2.1% of samples) that were from the same tank located in the Adelaide hills. The concentration of copper detected in the 8 samples ranged from 2.69 ppm to 3.47 ppm. The tank was plumbed-in and a filter installed, and the water used as drinking water.

### 3.2. Influence of Tank and Roof Material and Other Parameters on Metal Levels

Metal concentrations were compared with characteristics including tank and roof material, whether the tanks had first flush devices, the sampling corridor and the pH of the sample. The roof material was related to the levels of lead, zinc, copper, and cadmium (Table 2). An analysis of variance was used to show when the association was significant (*p* < 0.05). There was a relationship between lead and zinc and the sampling corridor, although this might be a result of the fact that the roof type in the Adelaide the hills foothills were predominately galvanised, whereas on the Adelaide plains, the roof types was predominately tiles. The presence of a first flush device had an influence on the level of cadmium and rainwater pH was related to the concentration of copper detected in water samples.

### 3.3. Seasonal Variability of Metals Load in Rainwater

There was no detectable trend between metal levels and the mean monthly rainfall in either the sampling month or the preceding month (Table 3). Adelaide’s wettest season extends from May to August, and early September [29]. In December 2015, and in February and April 2016, many tanks did not have water for sampling and so sampling across the three regions did not take place. For instance, 32 tanks (60.3%) in December 2015, 35 tanks (66%) in February 2016, and 28 tanks (52.8%) in April 2016 did not have enough water for sampling. Although the water was available in tanks for sampling, no sampling event occurred in July 2016.

### 3.4. Filters’ Capacity to Remove Metals from Rainwater

Nine tanks had filters fitted. As there were nine sampling events, a total of 72 paired samples (before and after filtration) were taken. Lead, copper and zinc were above the NHMRC guidelines in 29, 10, and 8 tanks, respectively. All (100%) of these samples remained above the NHMRC guidelines after filtration. A minor reduction in cadmium was observed with 8 samples above the guidelines before filtration and six samples after filtration. There was a statistically significant (*p* < 0.1) reduction in the concentration of all four metals (lead: *p* = 0.074, zinc: *p* < 0.05, copper *p* < 0.05, cadmium *p* < 0.05). However, this decrease was very small, and did not reduce any of the samples to less than the ADWG concentrations, except for two samples of cadmium.

## 4. Discussion

Lead was the metal of greatest concern detected in rainwater in the Adelaide region above the ADWG. The presence of lead in rainwater at concentrations above NHMRC guidelines is supported by previous studies. In 2010, a survey across South Australia found 62.8% of rainwater tanks to contain lead above NHMRC guideline levels. The highest concentration of lead record was 22.4 ppm [31]. Another study conducted across Australia found 79% of tanks to contain lead at levels exceeding guideline levels [32].

This study found that the concentration of lead in Adelaide rainwater ranged from <0.01 ppm (limit of detection) to 3.24 ppm, which is consistent with studies of other urban areas in Australia. In Brisbane (Queensland), a study detected lead in 15% of harvested rainwater samples at concentrations ranging from 0.01 ppm to 10.0 ppm (with one sample having a concentration of 85.0 ppm) [20]. In Sydney, Newcastle (New South Wales) and Esperance (Western Australia), the situation was similar. Water sampled from rain water tanks in Sydney contained lead up to 0.35 ppm [22], up to 0.16 ppm in Esperance [23], and up to 5.77 ppm in Newcastle [24]. A tank in the town of Karumba in the Shire of Carpentaria, northern Queensland contained up to 100 ppm lead [14]. These results demonstrate a need for future epidemiological studies to determine whether there is a public health risk from these detected levels.

In Adelaide, it is not clear where the lead found in rainwater samples comes from, although the relationship detected between lead and both roof material and geographic region might provide some clues. It is possible that the lead comes from the roof material, although lead as a component of galvanized or coated metal roofs is not regularly reported. The lead might be environmental. Vehicles were switched to unleaded petrol in 1985 [33], although it may take many decades for the metal to be removed from the environment. The NHMRC [34] indicates that in areas with history of higher road traffic, lead spread by vehicles before the introduction of unleaded petrol can still exist in roadside soils and, from there, the metal can spread to other areas in the environment [33]. Domestic paints in South Australia were reduced to less than 0.1% in 1997 [35], although it is possible this could still be a source of lead. Studies indicate that lead naturally occurs in the environment [36,37]. A study by Lovering [38] indicated that industrial precincts in large cities can have average atmospheric lead levles up to 2.5 µg/m^3^ or 0.0025 ppm, and that lead trace particles can spread in the environment miles away from the initial point of emission. Alternatively, lead could have been sourced by plumbing fixtures of plumbed-in tanks in older houses built before 1990 with galvanised pipes, chrome-plated brass, or piping joints sealed by adhesive that contained lead [39]. This could have been the case in the Adelaide Hills and foothills where tanks are essentially installed on older houses, and in the Adelaide plains where samples were mainly collected in semi-industrial areas. This is supported by a study in Brisbane that found lead above NHMRC guideline levels in 15% of rainwater samples and attributed lead paints and flashing to 79% of this contamination [40].

Fewer samples of zinc were over the ADWG. As with lead, there was a relationship between zinc and both roof material and geographic regions. This is more likely to come from galvanized roofing [4]. Zinc was higher in samples collected in the Adelaide hills and foothills, which are also those areas that have higher numbers of galvanized roofing. It is possible that lead and zinc found in rainwater could be linked to a combined corrosive action by solar radiation, wind, weathering, and pollution on rooftop structure materials. Studies indicate that an exposure to solar ultraviolet radiation can rapidly fade coatings on structure materials, scale off tiny metallic micro-particles and paints on coated surfaces, and trigger corrosion [41,42]. With rainfall, scaled off metallic particles can be easily removed from faded surfaces and get washed into the tank.

Cadmium and copper detection above the ADWG was limited to a few samples collected in the Adelaide hills. Copper was detected in samples collected from plumbed-in tanks, and the water used as a source of drinking water. Copper was the only metal that was influenced by the water pH and copper was only detected in those samples that had a pH ≤ 6.5 and is likely to be a result of corrosion of pipes. Acidic rainwater can result in increased corrosion and dissolve metals on tanks and roof materials structures, and on pipes, structure materials, and tank fittings [18,43]. Such water would be corrosive to copper and lead [44]. Another possible source of copper might be copper-chromium-arsenate (CCA)-treated timber. It should be noted that the Adelaide hills region was affected by extensive bushfires in January 2015 that caused damages to building structures, livestock, and to vineyard farms [45,46]. Few tanks had elevated levels of cadmium, and only in samples from the Adelaide hills. It is not clear where this cadmium might come from, although studies have reported that cadmium as a zinc impurity occurs in substantial amounts in galvanised structures [47].

### 4.1. Filters to Improve Rainwater Quality

A number of household filters are commercially available and most claim to remove a wide range of impurities, including metals and bacteria [48,49]; however, it was clear from this study that there was very little reduction in metals by the filters, even though there was a statistically significant reduction, the filters did not reduce contamination below the ADWG, except in two cases.

### 4.2. Exposure Assessment to Metals from Rainwater Harvested in the Adelaide Region

The exposure assessment is based on the proportion of samples that exceeded the ADWG thresholds, and the estimated proportion of households in Adelaide and regional South Australia that use rainwater as their primary source of drinking water. There are several studies which have previously examined the number of households in South Australia that are using rainwater for drinking. A survey conducted in 2015 and 2016 found that 36.4% (167/459) of household’s used rainwater as a main source of drinking water. The participants of this survey were from a range of areas across South Australia, including Adelaide [20]. This supports a previous study conducted in 2010 that found nearly 22% of South Australia households used rainwater as a source of drinking water, compared with the national average of 10.1% [4]. Adelaide has the highest proportion of households (10.6%) that use rainwater as a primary source of drinking water, compared to other capital cities [50]. In regional South Australia (outside of Adelaide), nearly 66% of households used rainwater as a source of drinking water [51].

**Figure 3:**
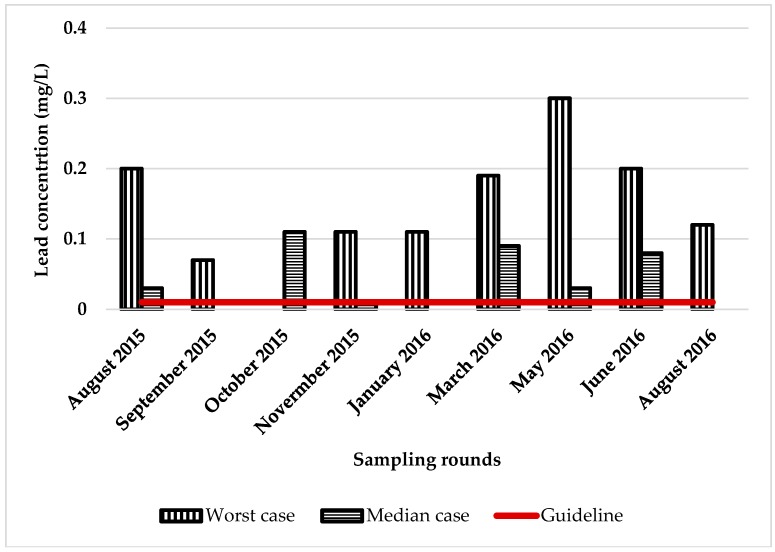
Levels of lead in the worst and median tanks.

**Figure 4:**
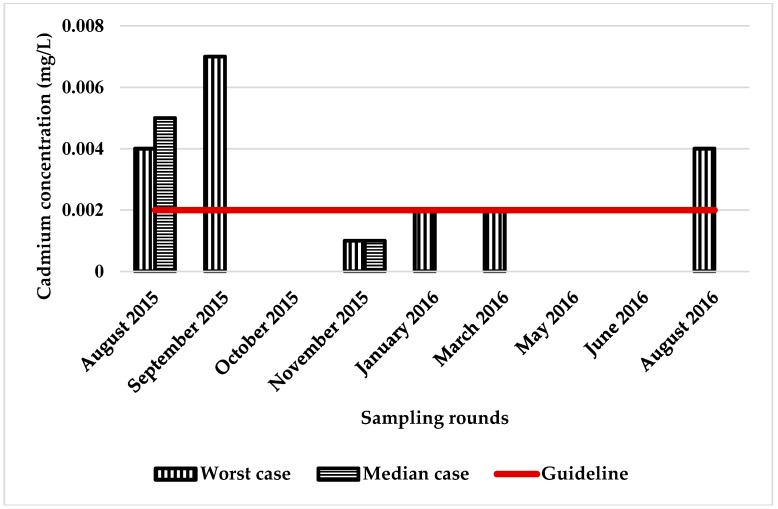
Levels of cadmium in the worst and median tanks.

**Figure 5:**
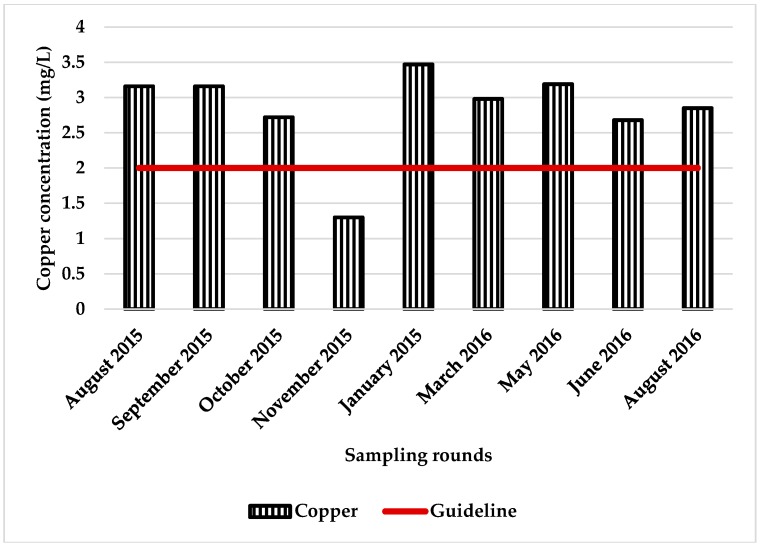
Levels of copper in the one tank found to have cooper above the ADWG.

Exposure was estimated using the median tank value for lead and cadmium compared with the ADWG (Figure 3 and Figure 4). Also included in these figures is the tank with the highest concentrations of lead and cadmium detected in the study (the worst case). In the worst case scenario, lead was found above the ADWG in one tank eight times in nine sampling rounds and, in the median scenario, five times in nine sampling rounds. Cadmium was found in one tank three times in nine sampling rounds in the worst case, and one time in nine sampling rounds in the median case scenario. Only one tank contained copper, at above the ADWG (eight times/nine sampling rounds), and this water was used as the primary source of drinking water (Figure 3, Figure 4 and Figure 5). If we assume that the median tank represents tanks across Adelaide, then at any one time approximately 6% of households are drinking water that exceeds the ADWG for lead. If the median tank represents tanks across South Australia, then in rural areas the estimate is much higher. This is consistent with an earlier study on rainwater across Australia, which found that 30/38 tanks surveyed (79% of tanks) contained lead above the ADWG [32].

The Australian public is advised not to use water with a lead concentration of 2.0 ppm or greater for garden watering as the metal can be taken up by the plants [52]. In total, seven samples (2% of samples) exceeded this limit. The FAO [53], and South Australia Environment Protection Authority (EPA) [54] set 0.10 ppm (0.1 mg lead/L) as the upper limit of lead concentration in livestock drinking water. In total, 100 samples (27.3% of samples) out of 365 samples tested for lead exceeded this limit. In the United States, a lower concentration is recommended for livestock feeding as it is argued that lead effects may occur from 0.05 ppm [55]. Epidemiologists argue that there is no safe threshold for lead in humans [56].

Cadmium detection above the ADGW in this study was limited to a few tanks in the Adelaide hills. In the median case scenario, 1.2% of Adelaide households are likely to be exposed to elevated blood cadmium levels. As for copper, the metal was detected above the ADWG in only one tank and, thus, no exposure assessment was conducted. However, it is important to note that this one tank was being used for drinking purposes and the concentration of copper detected (2.69 ppm to 3.47 ppm) was not only above guidelines for drinking water, but also guidelines for watering of plants and for poultry or sheep feeding [54].

## 5. Conclusions

This study indicates that lead was the metal detected in samples above the ADWG most often. Of 53 tanks surveyed, lead was detected in 47 tanks above the ADWG, in at least one sampling event. Zinc, cadmium, and copper were detected in fewer samples, predominantly in the Adelaide hills and foothills. Lead and zinc in rainwater content was consistent with roof materials and geographic area, although it was not possible to determine which of these effects was the primary contributor, as roof material in the Adelaide hills and Adelaide foothills are primarily, or solely, galvanised metal. In the absence of effective rainwater filtration systems, it is suggested that rainwater use be limited to non-potable purposes in areas where municipal water is supplied. However, there is a need for future studies investigating the potential human health impact of metal contamination to determine whether there is a public health risk from these levels.

## Figures and Tables

**Table 1:** Metal levels found in 365 rainwater samples and the number of tanks those samples came from.

**Lead**				
Level of contamination	Number of samples (out of 365)	Percentage (%)	Number of tanks (out of 53)	Percentage (%)
<0.01 ppm	185	50.6	10	18.8
0.01–0.10 ppm	83	22.7	15	28.3
0.11–1.0 ppm	84	22.9	19	35.8
>1.0 ppm	13	3.5	9	16.9
**Zinc**				
Level of contamination	Number of samples (out of 365)	Percentage (%)	Number of tanks (out of 53)	Percentage (%)
<3.0 ppm	312	85.4	24	45.2
3.1–4.0 ppm	24	6.3	15	28.3
4.1–5.0 ppm	16	4.3	8	15.0
>5.0 ppm	13	3.5	6	11.3
**Cadmium**				
Level of contamination	Number of samples (out of 365)	Percentage (%)	Number of tanks (out of 53)	Percentage (%)
<0.002 ppm	346	94.7	40	75.4
>0.002–0.003 ppm	9	2.4	7	13.2
>0.003–0.004 ppm	4	1.0	2	3.7
>0.004 ppm	6	1.6	4	7.5

**Table 2:** Relationships between metal concentrations and various parameters.

	Lead	Zinc	Copper	Cadmium
Tank material (galvanised steel and polyethylene)	0.696	0.335	0.228	0.846
Roof material (galvanised steel and tiles)	**0.001**	**0.015**	**0.001**	**0.004**
First flush installed	0.173	0.421	0.963	0.086 *
Corridor (Adelaide hills, foothills and plains)	**1.04 × 10^−5^**	**0.001**	0.825	0.608
Rainwater pH (ranged from pH 4.5 to pH 8.4)	0.681	0.823	0.074 *	0.950

(bold = *p* < 0.05, * = *p* < 0.1).

**Table 3:** Sampling rounds and lead and zinc levels in rainwater samples.

Sampling Year	Sampling Month	Metals in Tanks above the NHMRC Guideline		
		Number of Tanks out of 53 with Lead above NHMRC Guidelines (%)	Number of Tanks out of 53 with Zinc above NHMRC Guidelines (%)	Rainfall (mm) *
2015	August	31 (58.4)	3 (5.6)	67.8
	September	18 (33.9)	5 (9.4)	59.6
	October	16 (30.1)	7 (13.2)	41.9
	November	19 (35.8)	6 (11.3)	29.5
	December	N/A	N/A	29.1
2016	January	16 (30.1)	5 (9.4)	29.1
	February	N/A	N/A	15.6
	March	14 (26.4)	6 (11.3)	26.8
	April	N/A	N/A	39.0
	May	12 (22.6)	8 (15.0)	60.8
	June	17 (32.0)	7 (13.2)	77.0
	July	N/A	N/A	76.5
	August	22 (41.5)	6 (11.3)	77.7

* Mean monthly rainfall, Kent Town, Adelaide: 34.92° S, 138.62° E, station number: 23090 [30]. N/A = No samples collected.
